# The Effect of Long-Term or Repeated Use of Antibiotics in Children and Adolescents on Cognitive Impairment in Middle-Aged and Older Person(s) Adults: A Cohort Study

**DOI:** 10.3389/fnagi.2022.833365

**Published:** 2022-03-23

**Authors:** Zhou Liu, Shouchao Wei, Xiaoxia Chen, Lingying Liu, Zhuangsheng Wei, Zhimin Liao, Jiayuan Wu, Zhichao Li, Haihong Zhou, Duolao Wang

**Affiliations:** ^1^Guangdong Key Laboratory of Age-Related Cardiac and Cerebral Diseases, Department of Neurology, Institute of Neurology, Affiliated Hospital of Guangdong Medical University, Zhanjiang, China; ^2^Department of Neurology, Central People’s Hospital of Zhanjiang, Zhanjiang, China; ^3^Department of Neurology, Chenzhou No. 1 People’s Hospital, Chenzhou, China; ^4^Department of Neurology, Huizhou Municipal Central Hospital, Huizhou, China; ^5^Department of Clinical Research, Affiliated Hospital of Guangdong Medical University, Zhanjiang, China; ^6^Department of Clinical Sciences, Liverpool School of Tropical Medicine, Liverpool, United Kingdom

**Keywords:** antibiotics, children, adolescents, cognitive impairment, middle-aged and older person(s), cohort study

## Abstract

**Objectives:**

We evaluated the effects of long-term/recurrent use of antibiotics in childhood on developing cognitive impairment in middle and old age from UK Biobank Database.

**Methods:**

UK Biobank recruited participants aged 37–73 years. Cognitive impairment was ascertained by fluid intelligence questionnaire. Primary outcome was the occurrence of cognitive impairment in middle and old age. Multivariate logistic regression models were used to explore the relationship between long-term/recurrent use of antibiotics and cognitive impairment.

**Results:**

Over 3.8–10.8 years’ follow-up, 4,781 of the 35,921 participants developed cognitive impairment. The odds of cognitive impairment in middle and old age among long-term/recurrent use of antibiotics in childhood were increased by 18% compared with their counterparts (adjusted odd ratio 1.18, 95% confidence interval 1.08–1.29, *p* < 0.01). The effect of long-term/recurrent use of antibiotics in childhood on cognitive impairment was homogeneous across different categories of various subgroup variables such as sex, age, APOE4, ethnic groups, income before tax, smoking status, alcohol status, BMI, hypertension and diabetes but the effect of long-term/recurrent use of antibiotics in childhood was modified by the educational qualification (*p-*value for interaction <0.05).

**Conclusion:**

Long-term/recurrent use of antibiotics in childhood may increase the risk of cognitive impairment in middle and old age.

## Introduction

Cognitive impairment is the nomenclature of one or more cognitive impairment areas, ranging from complete cognition to mild cognitive impairment and finally dementia (Courtney, 2013; Ljubenkov and Geschwind, 2016; Rajji, 2018). Cognitive impairment is a serious health problem with high incidence, high disability rate and increasing global costs (Sacuiu, 2016). It is estimated that about 47 million people worldwide suffered from dementia in 2015, and the figure is expected to double every 20 years (Fiest et al., 2016; Livingston et al., 2017). This number will reach 66 million by 2030 and 115 million by 2050 (Fiest et al., 2016; Livingston et al., 2017). A growing body of evidence shows that age, cerebrovascular disease, diabetes, mental illness, etc., are the risk factors of cognitive impairment (Baumgart et al., 2015; Bellou et al., 2017).

Adolescence is a critical period of development, marking the transition from childhood to adulthood. It is in the last stage of development before adulthood that the brain is highly responsive to certain environmental cues (Paus et al., 2008). Compared with adults, the microbiota composition of adolescence is generally simpler and less stable, which is highly diverse and stable (Fouhy et al., 2012; Borre et al., 2014). These differences may be due to the relatively immature intestinal flora during adolescence, making it more susceptible to infection (Fouhy et al., 2012; McVey Neufeld et al., 2016). Therefore, significant changes in the composition of the gut microbiota may lead to the occurrence of neurodegenerative disorders (Verdu, 2006; McVey Neufeld et al., 2016). The use of antibiotics is an important reason for changes in the intestinal flora.

Antibiotics are usually used to remove or prevent bacterial colonization in the human body, and not to target specific types of bacteria (Hadar et al., 2018). Studies have found that antibiotic treatment impairs gut microbiota-brain communication and even cognitive impairment (Fröhlich et al., 2016). Slykerman et al. (2017) identified an association between antibiotic treatment in the first year of life and cognitive, behavior and emotion in childhood. However, there are limited clinical data on the effects of long-term/recurrent use of antibiotics as child or teenager (LRUAC) on cognitive impairment in middle and old age. Thus, the relationship between the LRUAC and the potential risk of developing dementia remains unknown. In the present study, we used a national population data bank in the United Kingdom to explore the associations between LRUAC and cognitive impairment in middle and old age.

## Materials and Methods

### Study Design and Population

We used the UK Biobank Database to perform the population-based retrospective cohort study with a representative sample of 502,505 participants. The cohorts, with subjects aged 37–73 years, were established from 2006 to 2010 across England, Scotland and Wales. At baseline (between 2006 and 2010), a detailed assessment of their characteristics and a completed cognitive testing were made. The participants were followed until 2014–2015. The study was approved by the North-West Multi-center Research Ethics Committee. The research was carried out in accordance with the Declaration of Helsinki of the World Medical Association, and participants gave informed consent. The data were anonymized and no additional ethical approval was required for the present analyses.

### Assessment of Cognitive Impairment

Fluid intelligence (FI) is defined as the ability to reason and to solve new problems independently of previously acquired knowledge, and is related to working memory (Salthouse and Pink, 2008), attention (Cochrane et al., 2019), executive functions (Lyall et al., 2016). FI is used as a tool to evaluate cognitive function (Kindermann et al., 2012; Kendall et al., 2019; Tank et al., 2022). Cognitive impairment was defined as >1 standard deviation (SD) reduction in FI score (Folley et al., 2019). FI score sums the number of correct answers out of 13 verbal and numerical reasoning items that participants were required to answer within 2 min. Baseline assessment of FI was carried out with touch-screen questionnaire in 2006–2010 in UK Biobank Assessment Centre, and follow-up assessment of FI was completed online follow-up remotely in 2014–2015. Primary outcome is the cognitive impairment in middle and old age.

### Assessment of Long-Term/Recurrent Use of Antibiotics as Child or Teenager

Long-term/recurrent use of antibiotics as child or teenager was recorded as “During childhood or as a teenager did you receive long-term or recurrent courses (3 or more per year) of antibiotics (for example for tonsillitis or acne)?” The above information was collected in 2017–2018.

### Assessment of Covariates

To control for confounding factors, we included the following baseline variables (2006–2010) as covariates in the analysis: age, sex, educational qualification, apoe4, ethnicity, income before tax, smoking status, alcohol status, Body Mass Index (BMI, classified as underweight: BMI ≤18.5, normal weight: BMI 18.5–24.9, overweight: BMI 25–29.9, and obesity: BMI ≥30.0). History of hypertension was originated from the question: “Has a doctor ever told you that you have had any of the following conditions?” And answers were: 1. Heart attack, 2. Angina, 3. Stroke, 4. High blood pressure, −7. None of the above, −3. “Prefer not to answer.” History of diabetes was derived from the question “Has a doctor ever told you that you have diabetes?”

### Statistical Analyses

Using the analysis of Chi-square tests or Fisher’s exact test, we compared the proportion of cognitive impairment by sociodemographic factors (e.g., gender, age, educational qualification, ethnicity, and income level), and clinical conditions and medical histories (e.g., Apoe4, smoking history, drinking history, BMI, history of hypertension, and diabetes). Using multivariate logistic regression models, we calculated adjusted odds ratios (ORs) and 95% confidence intervals (CIs) between LRUAC and non- LRUAC controlling for gender, age, qualification, apoe4, ethnicity, income level, smoking history, drinking history, BMI, history of hypertension, history of diabetes.

*P*-values less than 0.05 were considered statistically significant. Missing data in covariates were imputed using multiple imputation method (mice package, *m* = 5, method = “rf,” seed = 12). Sensitivity analysis was performed based on the complete cases without multiple imputation for missing values in covariates. Analyses were conducted with RStudio (Version 1.4.1717, PBC).

## Results

**FIGURE 1:**
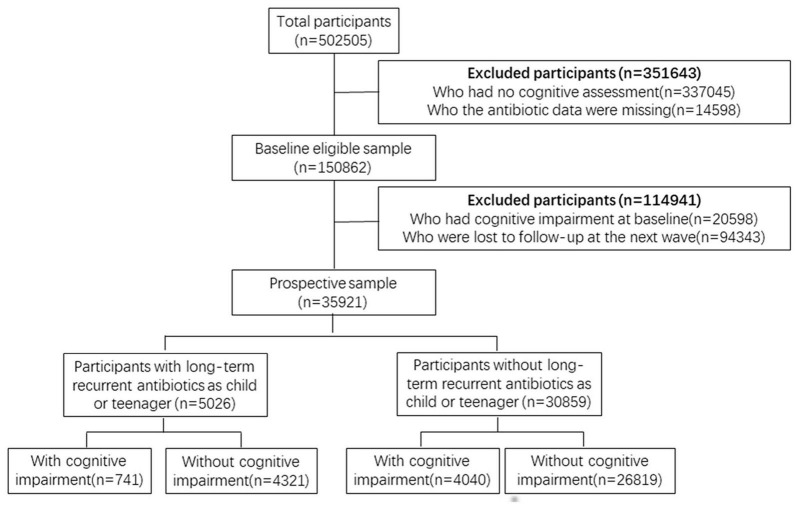
Flow chart of participant inclusion and grouping.

A total of 502,505 consecutive cases from UK Biobank were evaluated. 466,584 participants were excluded from the analysis because they had no cognitive assessment, they had cognitive impairment at baseline, they were lost to follow-up at the next wave or the antibiotic data were missing. After exclusions, a total of 35,921 participants without cognitive impairment remained in the study, which included 30,859 participants without long-term recurrent antibiotics as child or teenager. During an average 5.2 years’ follow-up (min 3.8, max 10.8 years), 741 (14.7%) participants with LRUAC and 4040 (13.1%) participants without LRUAC were identified cognitive impairment. Figure 1 shows the process of participant selection.

Table 1 presented potentially modifiable risk factors for cognitive impairment, included LRUAC, female, aged, low qualification, Ethnic groups, low income before tax, current smoking, non-alcohol, higher BMI, hypertension and diabetes.

**TABLE 1:** Demographic and clinical characteristics of patients at baseline.

Characteristics		Overall population (*n* = 35921)	Cognitive impairment		P
			yes	no	
LRUAC,%	Yes	35921	741 (14.6)	4321 (85.4)	**
	No		4040 (13.1)	26819 (86.9)	
Sex,%	Female	35921	2750 (13.6)	17462 (86.4)	
	Male		2031 (12.9)	13678 (87.1)	
Age,%	<60	35921	2434 (11.7)	18398 (88.3)	***
	60–70		2329 (15.5)	12665 (84.5)	
	> = 70		18 (18.9)	77 (81.1)	
Qualification,%	None of the below	35838	649 (37.2)	1098 (62.8)	***
	A levels/AS levels or equivalent; O levels/GCSEs or equivalent; CSEs or equivalent; Other professional qualifications		2759 (11.6)	21052 (88.4)	
	College or University degree; NVQ or HND or HNC or equivalent		1348 (13.1)	8932 (86.9)	
Apoe4,%	Yes	29926	1113 (13.5)	7130 (86.5)	–
	No		2826 (13.2)	18821 (86.8)	
Ethnic groups,%	White	35921	4421 (12.8)	30231 (87.2)	**
	Black		131 (26.3)	368 (73.7)	
	Asian		155 (34.1)	299 (65.9)	
	Other ethnic group		74 (23.4)	242 (76.6)	
Income before tax,%	Less than 18,000	32862	878 (21.5)	3201 (78.5)	***
	18,000 –51,999		2327 (13.8)	14580 (86.2)	
	52,000–100,000		788 (8.9)	8049 (91.1)	
	Greater than 100,000		223 (7.3)	2816 (92.7)	
Smoking status,%	Current	35868	381 (16.3)	1954 (83.7)	***
	Previous		1759 (13.9)	10897 (86.1)	
	Non		2635 (12.6)	18242 (87.4)	
Alcohol status,%	Current	35910	4468 (13.1)	29541 (86.9)	***
	Previous		126 (13.5)	813 (86.5)	
	Non		184 (19.2)	778 (80.8)	
BMI,%	Obesity	35261	1033 (15.1)	5828 (84.9)	***
	Overweight		1962 (13.5)	12567 (86.5)	
	Normal weight		1674 (12.3)	11982 (87.7)	
	Underweight		27 (12.6)	188 (87.4)	
Hypertension,%	Yes	35883	1219 (15.4)	6704 (84.6)	***
	No		3559 (12.7)	24401 (87.3)	
Diabetes,%	Yes	35882	203 (17.6)	951 (82.4)	***
	No		4569 (13.2)	30159 (86.8)	
Follow-up time (y)		35921	5.19 ± 1.02	5.29 ± 1.14	***

LRUAC: long-term/recurrent use of antibiotics in childhood. ^–^P > 0.05, **P < 0.01, and *** P < 0.001.

Table 2 displayed the relationship of LRUAC and cognitive impairment with logistic regression model. Compared with those without LRUAC, participants with LRUAC had a substantially higher odds for cognitive impairment by 20% (OR = 1.20; 95% CI, 1.10–1.31) after adjusting for Model 1 (age, sex, educational qualification, apoe4, and ethnic). The increased odds was slightly attenuated to 18% (OR = 1.18, 95% CI 1.08∼1.29) after adjusting for Model 2 (Model 1 plus income before tax, smoking status, alcohol status, BMI, hypertension, diabetes). The OR was 1.24 (95% CI 1.09–1.41) based on the complete cases without multiple imputation for missing values (Model 3) (Table 2, left). Other than that, after adjusting for Model 2, 60–70 years old, black, Asian, other ethnic group, current smoking increased the risk for cognitive impairment. While male, higher qualification and income, previous alcohol reduced the risk for cognitive impairment.

**TABLE 2:** Association between long-term/recurrent use of antibiotics and cognitive impairment and fluid intelligence (FI) difference.

	Cognitive impairment								
	Model 1 (*n* = 35921)			Model 2 (*n* = 35921)			Model 3 (*n* = 35921)		
	OR	95%CI	P	OR	95%CI	P	OR	95%CI	P
LRUAC (vs no)	1.20	1.10,1.31	**	1.18	1.08,1.29	**	1.24	1.09,1.41	**
Gender (vs. female)	0.93	0.87,0.98	*	0.89	0.83,0.95	***	0.94	0.87,1.02	
**Age (year) (vs.<60)**									
60–70	1.48	1.39,1.57	***	1.28	1.20,1.37	***	1.08	1.00,1.17	
> = 70	1.89	1.12,3.16	*	1.50	0.88,2.54		1.44	0.75,2.58	
Apoe4 (vs no)	1.02	0.95,1.10		1.03	0.95,1.11		1.05	0.96,1.14	
**Ethnic groups (vs White)**									
Black	2.68	2.18,3.28	***	2.69	2.19,3.30	***	2.53	1.99,3.22	***
Asian	3.90	3.20,4.76	***	4.05	3.30,4.96	***	4.10	3.22,5.21	***
Other ethnic group	2.17	1.67,2.82	***	2.17	1.66,2.83	***	2.35	1.71,3.26	***
**Qualification (vs None of the below)**									
A levels/AS levels or equivalent; O levels/GCSEs or equivalent; CSEs or equivalent; Other professional qualifications				0.26	0.24,0.30	***	0.31	0.27,0.36	***
College or University degree; NVQ or HND or HNC or equivalent				0.23	0.21,0.26	***	0.28	0.25,0.33	***
**Income before tax (vs Less than 18,000)**									
18,000–51,999				0.68	0.62,0.75	***	0.67	0.61,0.74	***
52,000–100,000				0.46	0.41,0.52	***	0.45	0.39,0.51	***
Greater than 100,000				0.38	0.32,0.44	***	0.35	0.29,0.42	***
**Smoking status (vs Non)**									
Current				1.26	1.12,1.43	***	1.21	1.04,1.40	***
Previous				1.06	0.99,1.13		1.07	0.98,1.15	
**Alcohol status (vs Non)**									
Current				0.88	0.74,1.05		0.89	0.72,1.11	
Previous				0.75	0.58,0.97	*	0.77	0.56,1.04	
**BMI (vs Underweight)**									
Obesity				1.17	0.77,1.78		1.17	0.73,2.01	
Overweight				1.11	0.73,1.69		1.20	0.74,2.05	
Normal weight				1.06	0.69,1.60		1.28	0.79,2.20	
Hypertension (vs no)				1.07	0.99,1.16		1.10	1.01,1.21	*
Diabetes (vs no)				1.32	0.95,1.32		1.05	0.86,1.28	

Analyses were adjusted for Model 1 (age, sex, qualification, apoe4, and ethnic groups); Model 2 (Model 1 plus income before tax, smoking status, alcohol status, BMI, hypertension, and diabetes); Model 3 (Model 2 based on completed cases without multiple imputation for missing values). LRUAC: long-term/recurrent use of antibiotics in childhood. *P < 0.05, **P < 0.01, and ***P < 0.001.

We also evaluated the risk of cognitive impairment after LRUAC in each variable subgroup and found that the effect of long-term/recurrent use of antibiotics in childhood on cognitive impairment was homogeneous across different categories of subgroup variables such as sex, age, APOE4, ethnic groups, income before tax, smoking status, alcohol status, BMI, hypertension and diabetes but the effect of LRUAC was modified by higher qualification (*p*-value for interaction <0.05, Table 3), with the decreased effect of LRUAC on cognitive impairment being found only among participants with a higher qualification of College or University degree; NVQ or HND or HNC or equivalent.

**TABLE 3:** Long-term/recurrent use of antibiotics and risk of cognitive impairment: Subgroup analysis.

	Number of subjects	OR,95%CI	P	OR,95%CI from interaction	P for interaction
**Sex**					
Female	20212	1.26,1.13–1.40	**	0.84,0.69–1.02	
Male	15709	1.04,0.88–1.22		1	
**Age**					
<60	20832	1.19,1.07–1.33	***	1	
60–70	14994	1.16,1.00–1.34	*	0.93,0.76–1.13	
> = 70	95	1.32,0.11–14.8		0.93,0.14–5.94	
**Qualification**					
None of the below	1747	1.62,1.19–2.19	**	1	
A levels/AS levels or equivalent; O levels/GCSEs or equivalent; CSEs or equivalent; Other professional qualifications	23811	1.16,1.04–1.30	**	0.73,0.53–1.03	
College or University degree; NVQ or HND or HNC or equivalent	10280	1.11,0.94–1.31		0.69,0.48–0.98	*
**Apoe4**					
No	26068	1.21,1.09–1.35	***	1	
Yes	9853	1.10,0.90–1.35		0.90,0.71–1.14	
**Ethnic groups**					
White	34652	1.20,1.10–1.31	***	1	
Black	499	0.83,0.46–1.50		0.78,0.44–1.38	
Asian	454	0.89,0.45–1.77		0.67,0.34–1.31	
Others	316	1.32,0.60–2.86		1.27,0.61–2.63	
**Income before tax**					
Less than 18,000	4525	1.34,1.07–1.68	*	1	
18,000–51,999	18568	1.10,0.96–1.26		0.85,0.65–1.12	
52,000–100,000	9555	1.16,0.94–1.43		0.91,0.68–1.23	
Greater than 100,000	3273	1.66,1.15–2.40	**	1.22,0.78–1.92	
**Smoking status**					
Non	20901	1.20,1.06–1.35	**	1	
Previous	12681	1.20,1.04–1.40	*	0.99,0.82–1.20	
Current	2339	0.99,0.72–1.36		0.86,0.61–1.21	
**Alcohol status**					
Non	963	1.23,0.76–1.99		1	
Previous	940	0.80,0.47–1.37		0.69,0.34–1.41	
Current	34018	1.20,1.09–1.31	***	0.95,0.58–1.56	
**BMI**					
Obesity	6979	1.20,1.01–1.44	*	1	
Overweight	14829	1.17,1.01–1.36	*	0.84,0.69–1.02	
Normal weight	13896	1.18,1.02–1.37	*	0.84,0.69–1.02	
Underweight	217	1.41,0.37–5.31		0.84,0.69–1.02	
**Hypertension**					
No	27991	1.21,1.10–1.34	***	1	
Yes	7930	1.09,0.91–1.31	0.43	0.95,0.76–1.17	
**Diabetes**					
No	34762	1.20,1.10–1.31	***	1	
Yes	1159	0.74,0.44–1.25		0.61,0.36–1.03	

Analyses were adjusted for age, sex, qualification, apoe4, ethnic group, income before tax, smoking status, alcohol status, BMI, hypertension, and diabetes. *P < 0.05, **P < 0.01, and ***P < 0.001.

## Discussion

In this population-based study, we found LRUAC was associated with higher risk of cognition impairment in middle and old age, independently of age, sex, educational qualification, apoe4, ethnic, income before tax, smoking status, alcohol status, Body Mass Index (BMI), history of hypertension, and history of diabetes.

Antibiotics changed the world and saved the lives of many patients who would have died of infection (Hadar et al., 2018). In fact, more than 10% of European children use antibiotics each year (Sturkenboom et al., 2008). In the United States, antibiotics account for 25% of all prescriptions for pediatricians (Finkelstein et al., 2003). However, a large body of evidence has begun to suggest that such frequent use of antibiotics can also come at a price. There is indeed evidence that antibiotic treatment has significantly changed the microflora composition of adults and infants (Francino, 2015; Azad et al., 2016; Yassour et al., 2016). Even short-term antibiotic treatment can have a long-term effect on the composition of microflora (Isaac et al., 2017). Stable microflora composition plays a role in regulating the immune system, hormone secretion and response, and metabolism (Spor et al., 2011; Neuman et al., 2015; Blaser, 2016; Shamriz et al., 2016). Therefore, changes in the composition of microflora caused by antibiotics may lead to disease. Exposure to antibiotics in young children is associated with an increased risk of excessive weight gain, asthma, allergies and autoimmune diseases, such as inflammatory bowel disease (IBD) (Cho et al., 2012; Cox et al., 2014; Bokulich et al., 2016; Korpela et al., 2016; Turta and Rautava, 2016). The impact of antibiotic use on future health and disease has caused widespread concern (Diamond et al., 2011; Diaz Heijtz et al., 2011).

Although no direct clinical studies prove that antibiotics are associated with cognition impairment, there are several cohort studies reveal effects of antibiotics on central nervous system diseases. Antibiotic exposure in the first 2 years of life increase risk of attention-deficit/hyperactivity disorder (ADHD) with average 8.8 years follow-up (Aversa et al., 2021). Even prenatal antibiotic exposure is associated with increased risk of ADHD (Hamad et al., 2020), cerebral palsy or epilepsy (Meeraus et al., 2015) in the child. Quinolone or d cephalosporins increase stroke risk slightly in older person(s) in 2 years (Luchsinger et al., 2001). In this study, we found LRUAC’s effect on cognition impairment more than 30 years later in middle and old age. These studies suggest that antibiotics could cause long-term brain damage. In this study, age, sex, apoe4, income before tax, smoking status, alcohol status, BMI, hypertension, and diabetes had no interaction with LRUAC but qualification (college or university degree; NVQ or HND or HNC or equivalent) had a quantitative interaction with LRUAC on cognitive impairment: the effect of LRUAC on cognitive impairment diminished in participants with high qualification, which is consistent with a previous study (Ko et al., 2021).

There are some animal studies exhibiting the effects of antibiotics on cognition and the mechanics in the brain. Lach’s study shows that microbiota depletion with antibiotic treatment during the adolescent period cause enduring neurobehavioral disfunction, with obvious change of gene expression in the amygdala (Lach et al., 2020). Study by Wang et al. (2015) showed that rats taking ampicillin increased serum corticosterone, increased anxiety-like behavior, and impaired spatial memory. Further support to this notion is the fact that antibiotics, such as streptozotocin, have been used to induce sporadic AD forms in animal models, thereby affecting learning and memory performance (Cattaneo et al., 2017). Antibiotic treatment from early adolescence significantly decreased hippocampal BDNF and enhanced learning and memory impairments in a mouse model of multiple sclerosis (Zeraati et al., 2019), and prevented the development of anxiety- and depression-related behaviors, oxidative stress and hypothalamic-pituitary-adrenal axis dysregulation in AD mice (Mosaferi et al., 2021). The evidence suggests that the use of antibiotic cocktails in adolescent mice can lead to permanent changes in intestinal microflora and an increase in pro-inflammatory cytokines (Desbonnet et al., 2014, 2015; Cattaneo et al., 2017). In humans, some antibiotics, cefepime, can cross the blood-brain barrier, leading to changes in mental state, loss of consciousness, myoclonus and insanity (Payne et al., 2017). fMRI study also shows that antibiotic influences insular cortex functional connectivity that could involve cognitive flexibility and memory processing (Sometti et al., 2021).

However, controversial results have been obtained in some clinical and animal trials. Studies have shown that antibiotics also have beneficial effects on Alzheimer’s disease. Through the triple eradication of antibiotics (omeprazole, clarithromycin, and amoxicillin) to eliminate Helicobacter pylori and other pathogens, the cognitive function parameters of patients with AD were positively improved (Kountouras et al., 2009). The possible mechanism is that some antibiotics can have a beneficial effect on Alzheimer’s disease by reducing neuroinflammation caused by biological disorders. Antibiotics have been shown to reduce inflammation and improve cognitive impairment in AD animal models. The use of rifampicin in the animal model of AD can reduce the levels of A β and inflammatory cytokines in the brain (Yulug et al., 2018). Minocycline has a similar effect on Aβ and reduces the activation of microglia in rodent AD model (Budni et al., 2016). In 2004, patients with suspected Alzheimer’s disease and mild to moderate dementia who took a combination of doxycycline and rifampicin significantly improved the cognitive subscale (SADAScog) of the Standard Alzheimer’s Disease Assessment scale at 6 months of age (Loeb et al., 2004). In contrast, in 2013, a multicenter, double-blind, randomized, 2 × 2 factor-controlled trial of mild to moderate AD patients showed no significant effect on cognitive ability after 12 months of treatment with doxycycline or rifampicin alone or in combination (Molloy et al., 2013). Similarly, in 1999, d-cycloserine was found to effectively improve cognitive impairment in patients with AD (Tsai et al., 1999), but these positive effects were not found in subsequent cochrane review (Laake and Oeksengaard, 2002).

The strengths of this study are the large sample size, the cohort study with long follow-up time, and the robust result for the effect of LRUAC on cognitive impairment. Also, the study has some limitations. Firstly, the records of LRUAC were collected during 2017–2018, which may be subject to recall bias. The cognition normal participants were more likely to recall they did not expose to LRUAC, which might exaggerate the effect of LRUAC. Secondly, the FI score was collected from different ways: basic collection in UK Biobank Assessment Centre during 2006–2010 and follow-up collection online remotely during 2014–2015, which would cause measurement bias toward the score. Lastly, this was an observational study and the observed results may still be subject to possible unobserved confounding factors.

In this population-based retrospective cohort study, we observed the impact of the LRUAC on the development of the cognitive impairment. We hope that our results could provide useful clues for assessing the lifelong effects of inappropriate use of antibiotics.

## Data Availability Statement

The original contributions presented in the study are included in the article/supplementary material, further inquiries can be directed to the corresponding authors.

## Ethics Statement

The studies involving human participants were reviewed and approved by the North-West Multi-center Research Ethics Committee. The patients/participants provided their written informed consent to participate in this study.

## Author Contributions

ZLiu: full access to the UK Biobank data and statistical analysis. HZ and DW: concept and design. ZLiu, SW, XC, and LL: drafting of the manuscript. All authors acquisition, analysis, or interpretation of data and critical revision of the manuscript for important intellectual content

## Conflict of Interest

The authors declare that the research was conducted in the absence of any commercial or financial relationships that could be construed as a potential conflict of interest.

## Publisher’s Note

All claims expressed in this article are solely those of the authors and do not necessarily represent those of their affiliated organizations, or those of the publisher, the editors and the reviewers. Any product that may be evaluated in this article, or claim that may be made by its manufacturer, is not guaranteed or endorsed by the publisher.
